# Determination of 11 minerals in children using inductively coupled plasma mass spectrometry

**DOI:** 10.1186/s12887-021-02962-z

**Published:** 2021-11-01

**Authors:** Xiaofeng Li, Chunnan Wang, Yang Wang, Xusheng Zhao, Na Li

**Affiliations:** 1 Children’s Neurorehabilitation Laboratory, Shenyang Children’s Hospital, 74 Chongshan Road East, Huanggu District, Shenyang, China; 2 Shenyang Children’s Hospital, Shenyang, China; 3 Key Laboratory of Shenyang Medical Mass Spectrometry Technology, Shenyang Harmony Health Medical Laboratory, Shenyang, China

**Keywords:** Minerals, Children, Reference values, Preventive healthcare, ICP-MS

## Abstract

**Background:**

Minerals play an important role in children’s growth and cognition. In this study, our aim was to establish reference intervals of mineral level in Children of Liaoning province (China) and provide a reliable basis for future preventive healthcare guidelines.

**Methods:**

Random samples of 2217 healthy children aged 0–15 years who were referred for routine hospital laboratory examinations in the cities of Shenyang, Fushun, Fuxin, Benxi, Chaoyang and Lingyuan were invited to participate in the study. Serum levels of Calcium (Ca), Iron (Fe), Zinc (Zn), Magnesium (Mg), Copper (Cu), Manganese (Mn), Selenium (Se), Mercury (Hg), Nickel (Ni), Cobalt (Co), and Lithium (Li) were determined by inductively coupled plasma mass spectrometer(ICP-MS).

**Results:**

Sex-related significant differences in the serum concentrations of the Mg and Cu (*P* < 0.05). Age-related significant differences were found in serum levels of Ca, Fe, Zn, Mg, Cu and Mn (*P* < 0.05) and the concentrations of 9 minerals (Ca, Mg, Cu, Mn,Se,Hg,Ni,Co and Li in serum varied from season to season (*P* < 0.05). The Spearman correlation matrix of 11 minerals was shown as a heat map, the correlations between Ca-Zn, Ca-Mg, Fe-Zn, Fe-Se, Mn-Ni, Se-Hg, Se-Ni, Hg-Ni, and Ni-Co, Ni-Li were the strongest compared with others.

**Conclusions:**

The reference intervals of serum 11 minerals for children should considering the sex, age and season, which may be useful for decisive diagnoses of abnormality of the 11minerals and the related diseases.

## Introduction

Minerals play a vital role in metabolism, tissue construction, enzyme systems, electrolyte balance in the body, and cell regeneration, and are essential nutrients to maintain human health as well [1, 2]. In particular, in the rapid growth of children, minerals are found to be of great significance [3]. They are not only essential substances for children’s growth and development, but also highly influence children’s cognitive development and immune system [4–6]. Therefore, detecting the levels of minerals in healthy children’s blood can provide a better nutrition and health guidance for scholars concentrating on the treatment of diseases caused by deficiency of essential minerals.

Reference interval (RI) is defined as the range between two thresholds, the results’ distribution of 2.5 and 97.5 percentages, within which 95% of healthy individuals. Establishing a reliable reference interval based on the levels of various minerals in healthy body can monitor people’s nutritional status. Due to the influences of different regions, environments, and lifestyles [7], the lack of a reliable reference for general population has still remained tangible. Different countries across the world have set their own references for specific populations, including Canada [8], Czech Republic [9, 10], Brazil [11], Germany [12], Italy [13, 14], etc. studies pointed out that differences in reference values are caused by overall differences in individuals’ age, sex, country of origin, food and drinking habits, smoking history, medication, etc. [11, 15, 16]. Geographic location was previously found as an important factor in determining levels of some minerals in the body, associating with geochemistry of soil and human health. A number of Chinese scholars attempted to establish general reference values for biological monitoring [17, 18], however, due to regional differences, reliable reference values for general population need to be further studied. To date, no epidemiological data have been reported regarding the status of minerals in healthy children in Liaoning province (China). Therefore, it is essential to acquire the levels of minerals in children living in the mentioned province through studies with large sample size, and propose the reference intervals, so as to provide a more comprehensive nutrition and health guidance

## Methods

### Study participants

Samples of 2217 healthy children aged 0–15 years old (1229 boys and 988 girls, boy: girl ratio, 1.24: 1) who underwent routine physical examinations were recruited from hospitals obtained by simple random sampling at Shenyang, Fushun, Fuxin, Benxi, Chaoyang and Lingyuan (Liaoning, China) from March 2019 to February 2020.

Inclusion criteria were the following:

1. no history of mellitus, hypertension, cardiovascular disease, liver disease, metabolism system, thyroid diseases, nutritional deficiency diseases, or other diseases affecting the concentrations of selected elements.
2. no specific mineral supplements were taken 3 month prior to the health examination of the children.

Exclusion criteria included malnutrition; overweight; obesity.

This study was approved by the Ethics Committee of Shenyang Children’s Hospital and the written informed consents were obtained from every child’s parents or other guardians. All the procedures involving human samples were carried out in accordance with the Declaration of Helsinki.

### Sample collection, preparation, and determination

Three days before the blood collection, all study participant maintained normal diet and exercise levels. At least 2 ml of venous blood was collected on an empty stomach, and the serum was isolated within 3 h (3500 rpm, 35 min), and frozen samples were then used for inspection. Samples with visible hemolysis, lipids, or jaundice were excluded from the analysis. One milliliter 5% HNO_3_ (Trace Metal Grade, Thermo Fisher, USA) solution was added to 0.5 ml serum sample to obtain a mixed solution, which was centrifuged at 12000 rpm for 5 min to obtain the supernatant. After that, 3.5 ml 1% HNO_3_ solution was added to 0.5 ml supernatant, and then the mixture was centrifuged for 2 min at 2500 rpm, and incubated at room temperature for 1 min to obtain the samples to be analyzed by ICP-MS (ICPMS-2030, Shimadzu, Japan). During the determination, accuracy and precision were checked by certified commercial element standard solution (1000 mg/L, the National Center of Analysis and Testing for Nonferrous Metals and Electronic Materials, Beijing, China).

### Statistics analysis

Descriptive data analysis was conducted, and date was expressed as median. Tukey’s test used to identify possible outliers (IQR: Q3-Q1; Q1: lower quartile, Q3: upper quartile). If the levels of value < Q1–3 IQR or > Q3 + 3 IQR, the value would be excluded as outlier. Reference intervals were based on the central 95% reference interval (P2.5-P97.5) of the data by using a nonparametric method. Mann-Whitney U test was used to indicate whether sex significantly affected the levels of minerals in the body. The differences in the concentrations of the serum minerals between different age groups and different seasons were further assessed using the Kruskal-Wallis test. To assess the correlation of minerals in the serum with age of different minerals, Spearman’s rank correlation test was performed. Partial correlation analysis was used to analyze the correlation of gender, age, season and minerals. Spearman correlation analysis was performed by programming in R language, and the correlation coefficient R value was used to reflect the correlation degree of 11 minerals. *P* < 0.05 was considered statistically significant. All statistical analyses were performed by using SPSS 22.0 software (IBM, Armonk, NY, USA). GraphPad Prism 5.0 software was used to draw line diagrams.

## Results

### Socio-demographic characteristics of the study participants

Among 2217 children (average age: 6.37 ± 3.99 years), there were 1229 (55.44%) males and 988 (44.56%) females. All participants were divided into five age-based groups:lactation (newborn to 1 year old, *n* = 160), infancy (1–3 years, *n* = 453), early childhood (3–6 years, *n* = 477), childhood (6–12 years, *n* = 909), adolescence (12–15 years,*n* = 218) (Table 1). The overall 95% reference intervals for serum levels of Ca, Fe, Zn, Mg, Cu, Mn, Se, Hg, Ni, Co, and Li were 80.40–104.00 mg/L, 0.40–2.05 mg/L, 515.00–1090.00μg/L, 16.30–24.30 mg/L, 663.43–1520.00μg/L, 0.00–1.34μg/L, 46.28–114.12μg/L, 0.04–0.83μg/L, 0.14–2.34μg/L, 0.07–0.73μg/L, 0.00–4.28μg/L, respectively (Table 2).

**Table 1:** Age and Sex Distributions in the Study Participants

Age (years)	Number of participants		
	Males(n)	Females(n)	Total
<1	82	78	160(7.22%)
1–3	254	199	453(20.43%)
4–6	278	199	477(21.52%)
6–12	466	443	909(41.00%)
12–15	149	69	218(9.83%)
Total	1229(55.44%)	988(44.56%)	2217(100%)

n = number of participants

The figure in the parentheses was the percentage of the total number of participants in each item

**Table 2:** 95% Reference Intervals of 11 Minerals in Different Sex

Element	Total(*n* = 2217)		Male(*n* = 1229)		Female(*n* = 988)		Z	*P*
	Median	[P2.5,P97.5]	Median	[P2.5,P97.5]	Median	[P2.5,P97.5]		
Ca	92.00	[80.40,104.00]	91.80	[80.30,104.00]	92.20	[81.10,104.00]	−1.229	0.242
Fe	1.04	[0.40,2.05]	1.04	[0.41,2.07]	1.04	[0.40,1.98]	− 0.029	0.977
Zn	783.00	[515.00,1090.00]	782.00	[512.00,1110.00]	784.00	[523.00,1087.10]	−0.269	0.921
Mg	20.20	[16.30,24.30]	20.30	[16.30,24.40]	20.00	[15.97,24.10]	−3.016	0.002
Cu	1020.00	[663.43,1520.00]	1034.00	[680.75,1541.00]	1010.00	[655.90,1510.00]	−4.192	< 0.001
Mn	0.39	[0.00,1.34]	0.40	[0.00,1.40]	0.39	[0.00,1.20]	−0.785	0.433
Se	78.70	[46.28,114.12]	78.70	[46.34,114.00]	78.80	[45.95,116.00]	−0.063	0.661
Hg	0.28	[0.04,0.83]	0.27	[0.03,0.81]	0.29	[0.05,0.90]	−2.043	0.041
Ni	0.92	[0.14,2.34]	0.94	[0.15,2.35]	0.90	[0.14,2.32]	−0.372	0.710
Co	0.31	[0.07,0.73]	0.31	[0.06,0.74]	0.31	[0.07,0.70]	−0.483	0.629
Li	1.17	[0.00,4.28]	1.19	[0.00,4.21]	1.15	[0.00,4.39]	−0.548	0.583

### Effects of sex on the concentrations of the 11 minerals

As shown in Table 2, Sex-related difference were statistically significant in Mg, Cu, Hg (*P* = 0.002, < 0.001 and 0.041, respectively) between boys and girls. After controlling the confounding factors by partial correlation analysis, there was sex correlation between Mn and Cu (Table 6). The 95% reference intervals for serum levels of Mg and Cu in different sex were 16.30–24.40 mg/L, 680.75–1541.00μg/L for boys and 15.97–24.10 mg/L, 655.90–1510.00μg/L for girls.

### Effects of age on the concentrations of the 11 minerals

As shown in Table 3, Except for Se, there were significant differences for other 10 elements in different age groups (*P* < 0.05) (Table 3). Ca, Mg, Cu, Mn, Hg and Li were negatively correlated with age. However, levels of Fe, Zn, Co increased gradually with age (Table 4), with significant differences present between the youngest (< 1 year old group) and the oldest (12-15 years old group) children. After controlling the confounding factors by partial correlation analysis, there was an age correlation between Ca, Fe, Zn, Mg, Cu and Mn (Table 6).

**Table 3:** The 95% Reference Intervals of 11 Minerals in Different Age-based Groups

Element	< 1 year old(*n* = 160)		1–3 years old(*n* = 453)		3–6 years old(*n* = 477)		6–12 years old(*n* = 909)		12-15 years old(*n* = 218)		F/χ2	*P*
	Median	[P2.5,P97.5]	Median	[P2.5,P97.5]	Median	[P2.5,P97.5]	Median	[P2.5,P97.5]	Median	[P2.5,P97.5]		
Ca	96.80	[86.03,108.96]	93.60	[83.73,104.90]	91.80	[81.10,102.12]	90.60	[78.98,102.33]	90.85	[81.00,101.34]	56.315	< 0.001
Fe	0.79	[0.30,1.80]	0.99	[0.37,2.24]	1.05	[0.40,2.06]	1.09	[0.46,1.99]	1.08	[0.48,2.29]	84.367	< 0.001
Zn	713.50	[492.15,1149.00]	748.00	[501.35,1050.00]	797.50	[523.93,1151.50]	796.00	[521.25,1092.50]	807.00	[543.45,1101.00]	10.981	< 0.001
Mg	21.25	[17.10,25.60]	20.50	[16.40,24.57]	20.10	[15.85,24.02]	19.80	[16.15,23.70]	20.20	[16.10,23.75]	19.939	< 0.001
Cu	864.50	[602.28,1299.75]	1060.00	[733.65,1596.75]	1100.00	[730.75,1600.00]	1030.00	[677.50,1460.00]	860.50	[607.60,1325.25]	260.176	< 0.001
Mn	0.48	[0.00,1.63]	0.43	[0.00,1.22]	0.38	[0.00,1.36]	0.39	[0.00,1.37]	0.36	[0.00,1.25]	20.140	< 0.001
Se	71.30	[35.43,119.00]	81.50	[48.95,118.93]	77.50	[47.32,112.67]	79.66	[50.87,111.00]	76.70	[55.06,116.20]	15.693	0.188
Hg	0.24	[0.00,0.70]	0.32	[0.01,1.08]	0.28	[0.05,0.86]	0.26	[0.05,0.74]	0.27	[0.05,0.68]	24.125	< 0.001
Ni	0.76	[0.24,2.61]	1.05	[0.16,2.51]	0.92	[0.13,2.31]	0.90	[0.15,2.31]	0.84	[0.10,2.53]	10.675	0.030
Co	0.27	[0.06,0.60]	0.31	[0.06,0.80]	0.33	[0.06,0.68]	0.31	[0.08,0.75]	0.32	[0.03,0.68]	24.755	< 0.001
Li	1.15	[0.00,4.73]	1.29	[0.00,4.38]	1.20	[0.02,4.08]	1.11	[0.00,4.46]	1.02	[0.00,3.86]	5.853	0.210

**Table 4:** Correlation Relationships between Serum Levels of 11 Minerals and Age

Element	Total		Male		Female	
	R	*P*	R	*P*	R	*P*
Ca	−0.26	< 0.001	−0.25	< 0.001	−0.28	< 0.001
Fe	0.18	< 0.001	0.18	< 0.001	0.18	< 0.001
Zn	0.13	< 0.001	0.13	< 0.001	0.15	< 0.001
Mg	−0.14	< 0.001	− 0.14	< 0.001	− 0.15	< 0.001
Cu	− 0.15	< 0.001	− 0.16	< 0.001	− 0.13	< 0.001
Mn	− 0.09	< 0.001	− 0.08	0.007	− 0.11	0.002
Hg	−0.07	0.001	−0.09	0.001	−0.04	0.226
Ni	−0.04	0.079	−0.15	0.609	−0.07	0.031
Co	0.05	0.040	0.04	0.164	0.06	0.110

### Effects of season on the concentrations of the 11 minerals

**Fig. 1:**
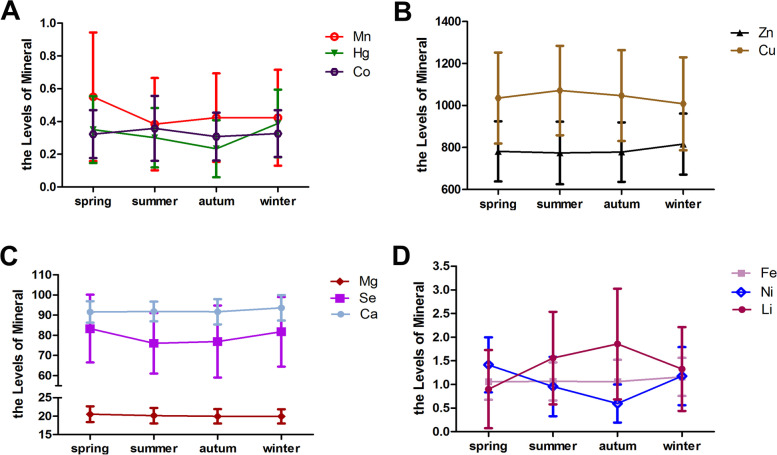
The difference of 11 minerals contents between seasons

As shown in Table 5, the concentrations of 11 minerals varied in different seasons (*P* < 0.001) (Table 5). After controlling the confounding factors by partial correlation analysis, Ca, Mg, Cu, Mn, Se, Hg, Ni, Co and Li had seasonal correlation (Table 6). Serum Co, Zn, Cu, Mg, Ca and Fe reference intervals showed stable trends within seasons, whereas serum Mn, Hg, Se, Ni and Li reference intervals demonstrated fluctuated trends with the change of seasons (Fig. 1).

**Table 5:** 95% Reference Intervals of 11 Minerals in Different Seasons

Element	Spring(*n* = 525)		Summer(*n* = 517)		Autumn(*n* = 619)		Winter(*n* = 554)		χ^2^	*P*
	Median	[P2.5,P97.5]	Median	[P2.5,P97.5]	Median	[P2.5,P97.5]	Median	[P2.5,P97.5]		
Ca	91.03	[81.20,102.26]	91.60	[82.80,102.00]	91.60	[78.85,103.57]	93.80	[79.88,105.03]	52.398	< 0.001
Fe	1.03	[0.40,1.91]	1.02	[0.43,2.01]	0.99	[0.35,2.32]	1.12	[0.44,2.05]	29.710	< 0.001
Zn	774.00	[516.20,1048.50]	777.00	[498.65,1110.00]	771.00	[513.50,1060.00]	814.00	[559.25,1111.25]	28.317	< 0.001
Mg	20.50	[16.32,24.90]	20.30	[16.10,24.30]	20.10	[16.25,23.80]	20.00	[16.40,23.91]	27.912	< 0.001
Cu	1020.00	[651.30,1510.00]	1060.00	[668.55,1500.00]	1030.00	[670.53,1566.85]	974.00	[656.75,1522.50]	29.449	< 0.001
Mn	0.47	[0.00,1.58]	0.34	[0.00,1.12]	0.39	[0.01,1.06]	0.40	[0.00,1.01]	55.970	< 0.001
Se	82.50	[47.74,117.75]	75.90	[47.95,107.05]	76.20	[42.41,115.20]	81.40	[48.05,118.25]	73.693	< 0.001
Hg	0.32	[0.03,0.89]	0.26	[0.07,0.81]	0.19	[0.02,0.71]	0.38	[0.07,0.97]	239.329	< 0.001
Ni	1.42	[0.26,2.60]	0.76	[0.11,2.34]	0.51	[0.10,1.57]	1.08	[0.21,2.52]	510.432	< 0.001
Co	0.34	[0.06,0.61]	0.32	[0.03,0.84]	0.28	[0.11,0.73]	0.32	[0.07,0.64]	22.028	< 0.001
Li	0.72	[0.00,3.08]	1.45	[0.12,3.99]	1.41	[0.54,4.89]	1.12	[0.18,4.33]	294.421	< 0.001

**Table 6:** Partial Correlation between Sex, Age and Season and 11Minerals

Characteristics	Ca	Fe	Zn	Mg	Cu	Mn	Se	Hg	Ni	Co	Li
Sex^a^	–	–	–	−0.08^**^	−0.09^***^	–	–	–	–	–	–
Age (years old)^b^	−0.25^***^	0.19^***^	0.13^***^	−0.13^***^	−0.19^***^	−0.08^**^	–	–	–	–	–
Season^c^	0.15^***^	–	–	−0.12^***^	−0.07^**^	− 0.16^***^	−0.16^***^	− 0.16^***^	−0.38^***^	− 0.08^**^	0.16^***^

^a^ Controlling for age, season, educational level of parents, and family economic level

^b^ Controlling for sexr,season, educational level of parents, and family economic level

^c^ Controlling for sex, age, educational level of parents, and family economic level

- Indicates that the correlation is not statistically significant

***P < 0.001; **P < 0.01; *P < 0.05

### Correlation among serum levels of 11 minerals

**Fig. 2:**
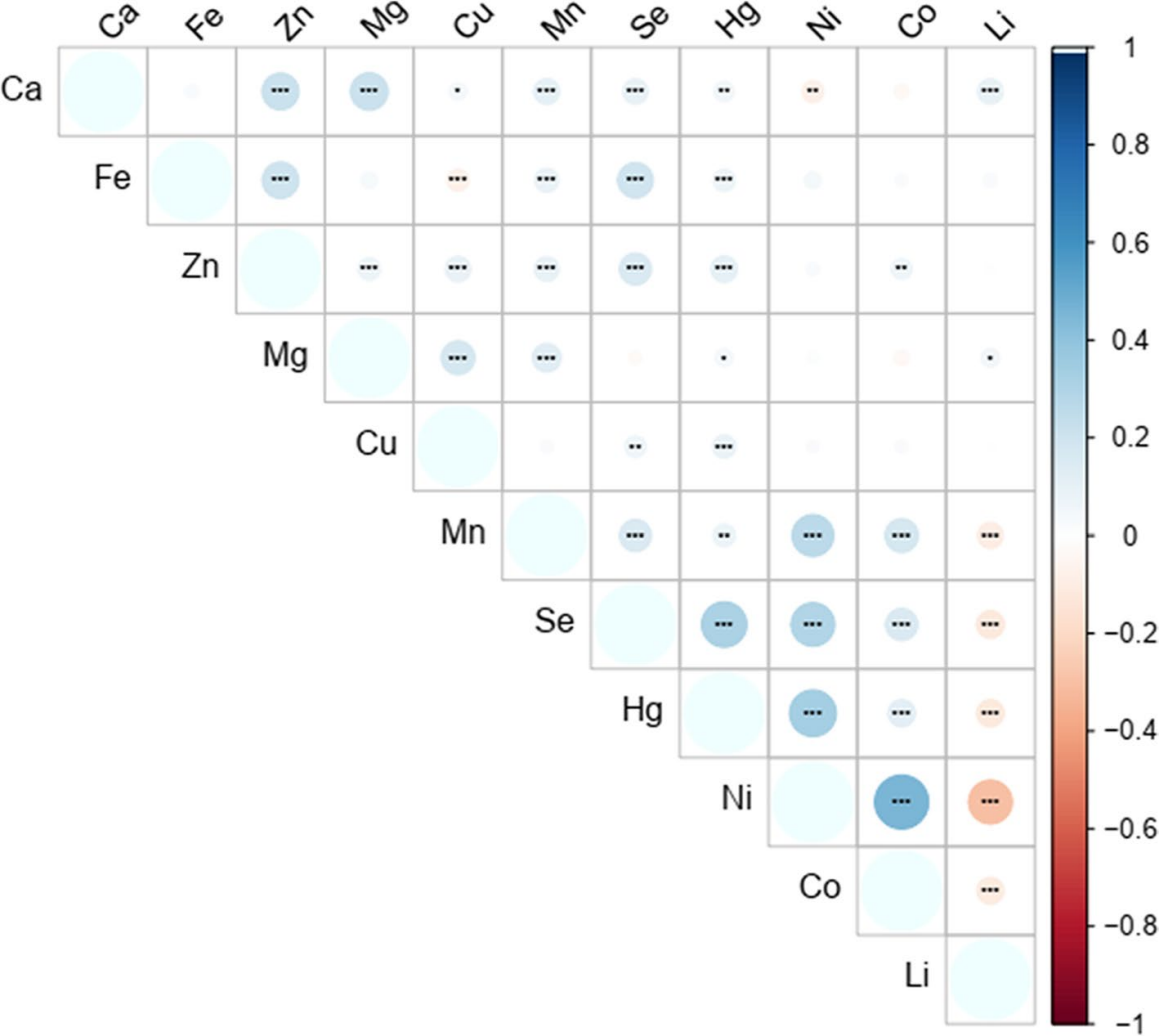
Heat map showing the Spearman’s correlation coefficients of serum levels of 11 minerals. The color corresponds to the strength of correlations (blue: positive correlation; white: no correlation; red: negative correlation),* *P* < 0.05, ** *P* < 0.01, *** *P* < 0.0001

The Spearman correlation matrix of 11 minerals of the 2217 participants was shown as a heat map on Fig. 2. Herein, a total of 41 significant correlations were found, and the majority of them were positive and only seven correlations were negative (Ca-Ni, Fe-Mn, Li-Mn, Li-Se, Li-Hg, Li-Ni, Li-Co). Correlations between Ca-Zn, Ca-Mg, Fe-Zn, Fe-Se, Mn-Ni, Se-Hg, Se-Ni, Hg-Ni, and Ni-Co were the strongest compared with others.

## Discussion

The background level of minerals in children’s body is of great significance for monitoring the environment and evaluating children’s nutritional status [15, 19, 20]. Our study established the reference intervals of serum Ca, Fe, Zn, Mg, Cu, Mn, Se, Hg, Ni, Co, Li for children in a large number of relatively healthy participants (Table 1), and make a more detailed classification considering the sex (Tables 2 and 6), age (Tables 3, 4 and 6) and season (Tables 5 and 6 and Fig. 1), which may be useful for diagnoses of abnormality of the 11 minerals and the related diseases.

This study revealed the existence of the sex-associated differences in serum levels of Mg, Cu in which Mg, Cu level was higher (*P* < 0.05). The existence of differences may be related to dietary habits, gastrointestinal digestion and absorption capacity, as well as physiological differences [21–23], while the exact mechanism detailing how the levels of these minerals vary based on sex has still remained obscure.

Many studies have indicated that the concentrations of minerals were closely related to age [24, 25]. The concentrations of serum Ca, Fe, Zn, Mg, Cu, Mn showed a noticeable age dependency in this study (*P* < 0.05), which consistent with previous studies [24–27]. It’s a common finding that babies who rely on breast milk or infant formula have higher levels of calcium in their blood and the lowest levels of iron and zinc. However, as the body develops rapidly, calcium deposition in the bone leads to a decrease in calcium levels under the action of osteoblasts, while iron and zinc levels increase with the addition of complementary food. Serum iron level affects the absorption of manganese. Therefore, when serum iron level increased, serum manganese level decreased. The change of children’s dietary structure with age may lead to the decrease or increase of other elements. However, the limitation of our study was that we did not consider the data of mineral content in diet or drink. Physical activities [28] and even air pollution [29] may also affect the concentrations of these minerals.

We also found that the concentrations of these 9 minerals (Ca, Mg, Cu, Mn,Se,Hg,Ni,Co and Li) in serum were affected by seasons, which were rarely mentioned in other studies, to our known. However, the reasons are still unknown. We speculate that Liaoning (N38°43′ ~ 43°26′, E118°53′ ~ 125°46′) is located in the south of Northeast China, with four distinct seasons of spring, summer, autumn and winter, the temperature here in spring and autumn is 8 ~ 10 and 15 ~ 19 °C, in summer and winter is 23 ~ 25 °C and-10 ~ − 17 °C, respectively. The change of temperature will cause people here to have different eating habits, patterns and activities in different seasons, these may be the reasons for the seasonal variation difference related to minerals. Parents pay more attention to the key role of trace elements in children’s growth. Therefore, it seems to be an attractive strategy and effective intervention for children’s health to strengthen or adjust the diet intake and to supplement or reduce the mineral supplement according to the season.

Correlations between essential trace elements have been studied in children previously [30–33]. However, the results are not always consistent. Our study found that significant correlations were found between the pairs of 41 trace elements. The positive correlation of Fe-Zn in blood has been previously reported, which may be because Fe and Zn have similar chemical properties and have the same electron shell structure. Studies have shown that both iron and copper could inhibit the absorption of Zn in diet [34, 35]. However, in our study, we found that there is a highly positive correlation between Fe-Zn and Cu-Zn. From birth to 2 years of age or even older, children in Liaoning are suggested to be supplemented with calcium, vitamin D, iron and zinc for prevention of rickets, anemia, and hypoimmunity. However, when parents supplement these elements, they are not aware of the interaction between these basic elements. We have found that there is a strong positive correlation between Fe-Zn, so the appropriate proportion of Fe-Zn may help to increase the absorption of these two elements. There is complex mutual promotion or inhibition between minerals. Therefore, attention should be paid to the content of essential trace elements when purchasing micronutrient products.

In many studies, atomic absorption spectrometry is the most widely used detection method, but it has the disadvantages of fewer detection elements, long detection time, narrow linear range, and high safety requirements for equipment. At present, ICP-MS is widely used for measuring the levels of minerals in blood, tissue, and body fluid for clinical nutrition or testing and monitoring of toxicity. The use of ICP-MS realized the measurement of very low concentrations of metals or metalloids in the biological liquids, and will gradually replace atomic absorption spectrometry. However, due to the different equipment and methods, the integration and transfer of electrolyte reference intervals need to be further verified.

The detection of minerals is influenced by several factors and remarkably varies in different regions. As a result, there are no uniform reference values in China, and various minerals have different corresponding reference values in different regions. Our data established reference intervals for serum levels of 11 minerals for children who lived in Liaoning province, and can provide guidelines for environmental monitoring, preventive health care and screening and diagnosis of nutritional diseases.

### Limitations

In this study, we tried our best to investigate to explore the related factors affecting serum minerals of healthy children in Liaoning, China, so as to provide reference for the formulation of reference intervals for serum minerals in children. However, our study also has some limitations. Children’s serum mineral level can only be obtained from one measurement, which can not reflect the long-term human mineral level. A larger sample size is recommended to find potential factors affecting children’s serum minerals and ensure the universality of the research results.

## Conclusion

Feasible pediatric minerals reference intervals are lacked in China, thus, we conducted this research to establish serum levels of Ca, Fe, Zn, Mg, Cu, Mn, Se, Hg, Ni, Co, and Li reference intervals to fill the gap. Our study found that the concentration of 11 minerals in children’s serum is related to sex, age and season. The study has provided further information on a wide range of serum 11 minerals of children as reference levels for Liaoning which are currently lacking, and is of great significance for the diagnosis of 11 mineral abnormalities and related diseases.

## Data Availability

The datasets used and/or analyzed during the current study are available from the corresponding author on reasonable request.

## Abbreviation

ICP-MS: inductively coupled plasma mass spectrometry
